# Supplementary material for: EGCG Disrupts the LIN28B/Let-7 Interaction and Reduces Neuroblastoma Aggressiveness
Source: Int J Mol Sci. 2024 Apr 27;25(9):4795. doi: 10.3390/ijms25094795 (PMC11084668; doi:10.3390/ijms25094795)
Supplement: Supplementary file 1 [file ijms-25-04795-s001.zip › ijms-2952221-supplementary.pdf]

Supplementary Materials

EGCG disrupts the LIN28B/Let-7 interaction and reduces neuroblastoma aggressiveness

Simona Cocchi [1], Valentina Greco [1,†], Viktoryia Sidarovich [1], Jacopo Vigna [1,2], Francesca Broso [1], Diana Corallo [3], Jacopo Zasso [1,‡], Angela Re [1,§], Emanuele Filiberto Rosatti [1], Sara Longhi [1], Andrea Defant [2], Federico Ladu [4], Vanna Sanna [5], Valentina Adami [1], Vito G. D'Agostino [1], Mattia Sturlese [6], Mario Sechi [4], Sanja Aveic [3], Ines Mancini [2], Denise Sighel [1,*,‖] and Alessandro Quattrone [1,*,‖]

1 Department of Cellular, Computational and Integrative Biology (CIBIO), University of Trento, 38123 Trento, Italy;
2 Department of Physics, University of Trento, 38123 Trento, Italy;
3 Istituto di Ricerca Pediatrica Fondazione Città della Speranza, 35127 Padova, Italy;
4 Department of Medicine, Surgery and Pharmacy, University of Sassari, Sassari 07100, Italy;
5 Nanomater S.r.l, Alghero 07041, Italy;
6 Molecular Modeling Section, Department of Pharmaceutical and Pharmacological Sciences, University of Padua, 35127 Padova, Italy;
* Correspondence: denise.sighel@unitn.it (D.S.); alessandro.quattrone@unitn.it (A.Q.); Tel.: +39-0461-283096 (D.S)
† Current address: UOM Anatomia e Istologia Patologica, Santa Chiara Hospital, APSS, 38123 Trento, Italy
‡ Current address: Human Technopole, 20157 Milan, Italy
§ Current address: Department of Applied Science and Technology, Politecnico di Torino, 10129 Torino, Italy
‖ These authors contributed equally to this work.

Contents: **Page(s):**

Contents	Page(s)
**Figure S1.** Purification and Functional binding of rLIN28B to pre-let-7g miRNA by AlphaScreen and REMSA.	**S2**
**Table S1.** Compounds selected following the primary and the confirmatory Alpha Screenings.	**S3-4**
**Figure S2.** EGCG stability evaluation by HPLC and assessment of EGCG-NP penetration and effect in NB cells.	**S5**
**Figure S3.** Empty-NP treatment only slightly affects NB cell proliferation, and EGCG-NP treatment increases let-7 miRNA levels in SK-N-BE(2) cells.	**S6**
**Table S2.** Primer sequences.	**S7**
**Table S3.** Protein purification: buffers composition.	**S7**

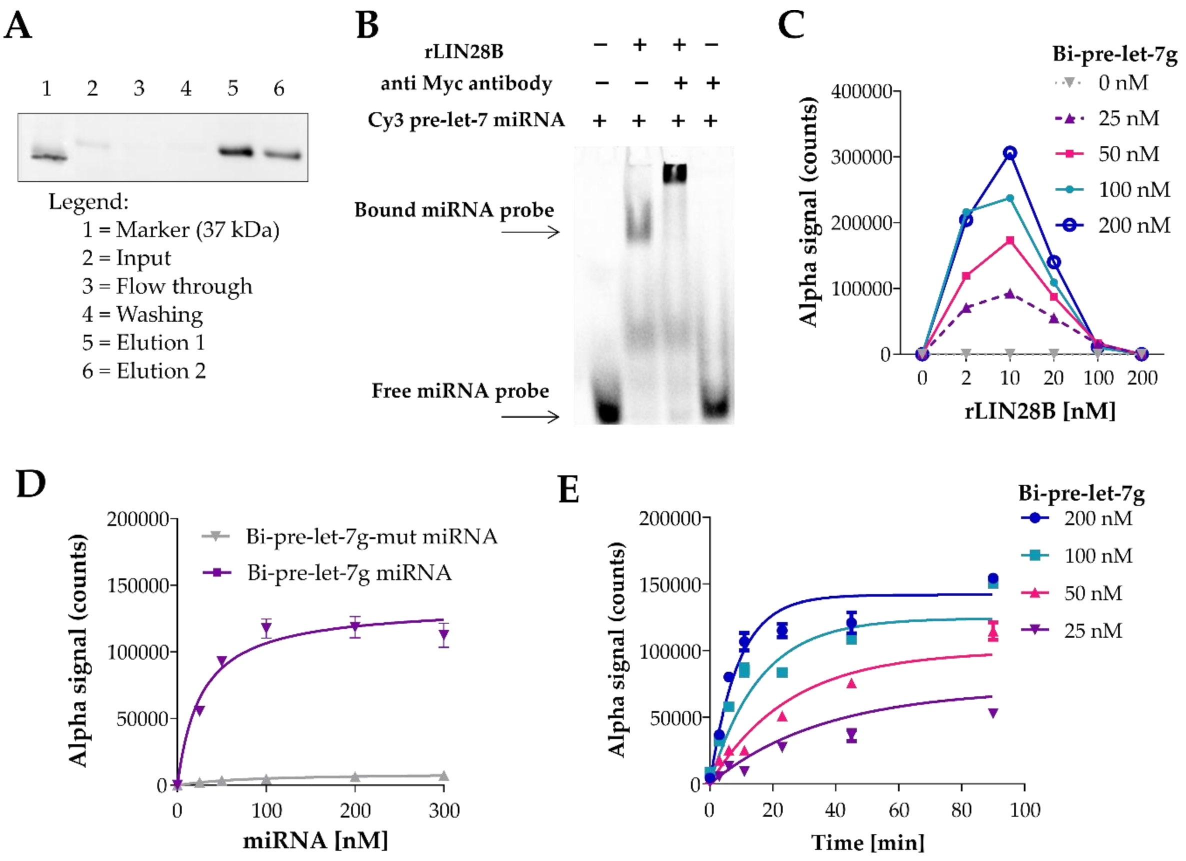

Figure S1. Purification and functional binding of rLIN28B to pre-let-7g miRNA by AlphaScreen and REMSA. (**A**) Immunoblot detection of His6-cMyc-tagged human recombinant LIN28B protein (rLIN28B) produced in HEK293T cells using a monoclonal anti-Myc antibody. (**B**) Representative REMSA results showing the formation of a rLIN28B/Cy3-labelled-pre-let-7g miRNA complex. The free Cy3-labelled-pre-let-7g miRNA probe alone is detected at lower molecular weights. Anti-Myc antibody addition in the binding reaction produces a complex supershift. (**C**) rLIN28B titration curves obtained with different concentrations of the biotinylated pre-let-7g miRNA probe performed to identify the 'hook' point of the AlphaScreen assay. (**D**) Saturation binding experiment to determine the apparent K*d* value for rLIN28Bprotein/biotinylated pre-let-7g miRNA interaction. K*d* = 34.7 ± 3.6 nM. n = 3 replicates. Mean ± SD. (**E**) The time course of the binding reaction showing that the binding of rLIN28B to the biotinylated pre-let-7g miRNA probe is both time and dose-dependent. n = 2 replicates. Mean ± SD.

Table S1. Compounds selected following the primary and the confirmatory Alpha Screenings.

ID	Compound Name	CAS number	Formula	Mol. Wt.	CANONICAL SMILES
**EGCG**	epigallocatechin-3-monogallate	989-51-5	$C_{22}H_{18}O_{11}$	458.38	C1C(C(OC2=CC(=CC(=C21)O)O)C3=CC(=C(C(=C3)O)O)O)OC(=O)C4=CC(=C(C(=C4)O)O)O
**TFMG**	theaflavin monogallates	30462-34-1 [3-isomer], 28543-07-9 [3′-isomer]	$C_{36}H_{28}O_{16}$	716.61	C1C(C(OC2=CC(=CC(=C21)O)O)C3=CC(=C(C4=C(C(=O)C=C(C=C34)C5C(CC6=C(C=C(C=C6O5)O)O)OC(=O)C7=CC(=C(C(=C7)O)O)O)O)O)O)O
**GA**	gallic acid	149-91-7	$C_7H_6O_5$	170.12	C1=C(C=C(C(=C1O)O)O)C(=O)O
**ATA**	aurin tricarboxylic acid	4431-00-9	$C_{22}H_{14}O_9$	422.35	C1=CC(=C(C=C1C(=C2C=CC(=O)C(=C2)C(=O)O)C3=CC(=C(C=C3)O)C(=O)O)C(=O)O)O
**1**	terbutaline hemisulfate	23031-32-5	$C_{12}H_{21}NO_7S$	323.37	CC(C)(C)NCC(C1=CC(=CC(=C1)O)O)O.CC(C)(C)NCC(C1=CC(=CC(=C1)O)O)O.OS(=O)(=O)O
**2**	thioguanine	154-42-7	$C_5H_5N_5S$	167.19	C1=NC2=C(N1)C(=S)N=C(N2)N
**3**	thioridazine hydrochloride	130-61-0	$C_{21}H_{27}ClN_2S_2$	407.04	CN1CCCCC1CCN2C3=CC=CC=C3SC4=C2C=C(C=C4)SC.Cl
**4**	suramin hexasodium salt	129-46-4	$C_{51}H_{34}N_6Na_6O_{23}S_6$	1429.19	CC1=C(C=C(C=C1)C(=O)NC2=C3C(=CC(=CC3=C(C=C2)S(=O)(=O)[O-])S(=O)(=O)[O-])S(=O)(=O)[O-])NC(=O)C4=CC(=CC=C4)NC(=O)NC5=CC=CC(=C5)C(=O)NC6=C(C=CC(=C6)C(=O)NC7=C8C(=CC(=CC8=C(C=C7)S(=O)(=O)[O-])S(=O)(=O)[O-])S(=O)(=O)[O-])C.[Na+].[Na+].[Na+].[Na+].[Na+].[Na+]
**5**	diflubenzuron	35367-38-5	$C_{14}H_9ClF_2N_2O_2$	310.69	C1=CC(=C(C(=C1)F)C(=O)NC(=O)NC2=CC=C(C=C2)Cl)F
**6**	*N*-hydroxymethylnicotinamide	3569-99-1	$C_7H_8N_2O_2$	152.15	C1=CC(=CN=C1)C(=O)NCO
**7**	salicylanilide	87-17-2	$C_{13}H_{11}NO_2$	213.24	C1=CC=C(C=C1)NC(=O)C2=CC=CC=C2O
**8**	dibutyl phthalate	84-74-2	$C_{16}H_{22}O_4$	278.35	CCCCOC(=O)C1=CC=CC=C1C(=O)OCCCC
**9**	aminosalicylate sodium	6018-19-5	$C_7H_6NNaO_3$	175.12	C1=CC(=C(C=C1N)O)C(=O)[O-].O.O.[Na+]

**10**	amoxicillin	26787-78-0	$C_{16}H_{19}N_3O_5S$	365.41	CC1(C(N2C(S1)C(C2=O)NC(=O)C(C3=CC=C(C=C3)O)N)C(=O)O)C
**11**	amphotericin B	1397-89-3	$C_{47}H_{73}NO_{17}$	924.10	CC1C=CC=CC=CC=CC=CC=CC=CC(CC2C(C(CC(O2)(CC(CC(C(CCC(CC(CC(=O)OC(C(C1O)C)C)O)O)O)O)O)O)O)C(=O)O)OC3C(C(C(C(O3)C)O)N)O
**12**	anthralin	480-22-8, 1143-38-0	$C_{14}H_{10}O_3$	226.23	C1C2=C(C(=CC=C2)O)C(=O)C3=C1C=CC=C3O
**13**	chloramphenicol	530-43-8	$C_{27}H_{42}Cl_2N_2O_6$	561.55	CCCCCCCCCCCCCCCC(=O)OCC(C(C1=CC=C(C=C1)[N+](=O)[O-])O)NC(=O)C(Cl)Cl
**14**	chlorcyclizine hydrochloride	1620-21-9	$C_{18}H_{22}Cl_2N_2$	337.30	CN1CCN(CC1)C(C2=CC=CC=C2)C3=CC=C(C=C3)Cl.Cl
**15**	dapsone	80-08-0	$C_{12}H_{12}N_2O_2S$	248.31	C1=CC(=CC=C1N)S(=O)(=O)C2=CC=C(C=C2)N
**16**	ethionamide	536-33-4	$C_8H_{10}N_2S$	166.25	CCC1=NC=CC(=C1)C(=S)N
**17**	telenzepine hydrochloride	80880-90-6	$C_{19}H_{23}ClN_4O_2S$	406.94	CC1=C2C(=CS1)C(=O)NC3=CC=CC=C3N2C(=O)CN4CCN(CC4)C
**18**	medroxyprogesterone acetate	71-58-9	$C_{24}H_{34}O_4$	386.54	CC1CC2C(CCC3(C2CCC3(C(=O)C)OC(=O)C)C)C4(C1=CC(=O)CC4)C
**19**	piperazine	110-85-0	$C_4H_{10}N_2$	86.14	C1CNCCN1
**20**	procaine hydrochloride	51-05-8	$C_{13}H_{21}ClN_2O_2$	272.78	CCN(CC)CCOC(=O)C1=CC=C(C=C1)N.Cl
**21**	acedapsone	77-46-3	$C_{16}H_{16}N_2O_4S$	332.38	CC(=O)NC1=CC=C(C=C1)S(=O)(=O)C2=CC=C(C=C2)NC(=O)C
**22**	doxorubicin	23214-92-8	$C_{27}H_{29}NO_{11}$	543.53	CC1C(C(CC(O1)OC2CC(CC3=C2C(=C4C(=C3O)C(=O)C5=C(C4=O)C(=CC=C5)OC)O)(C(=O)CO)O)N)O
**23**	dehydro (11,12)ursolic acid lactone		$C_{30}H_{46}O_3$	454.70	CC1CCC23CCC4(C5(CCC6C(C)(C(CCC6(C5C=CC4(C3C1C)OC2=O)C)O)C)C)C
**24**	coralyne chloride	38989-38-7	$C_{22}H_{22}ClNO_4$	399.88	CC1=C2C=C(C(=CC2=CC3=[N+]1C=CC4=CC(=C(C=C43)OC)OC)OC)OC.[Cl-]
**25**	2',5'-dihydroxy-4-methoxychalcone	6342-92-3	$C_{18}H_{18}O_6$	330.34	COC1=CC=C(C=C1)C=CC(=O)C2=C(C=CC(=C2)O)O

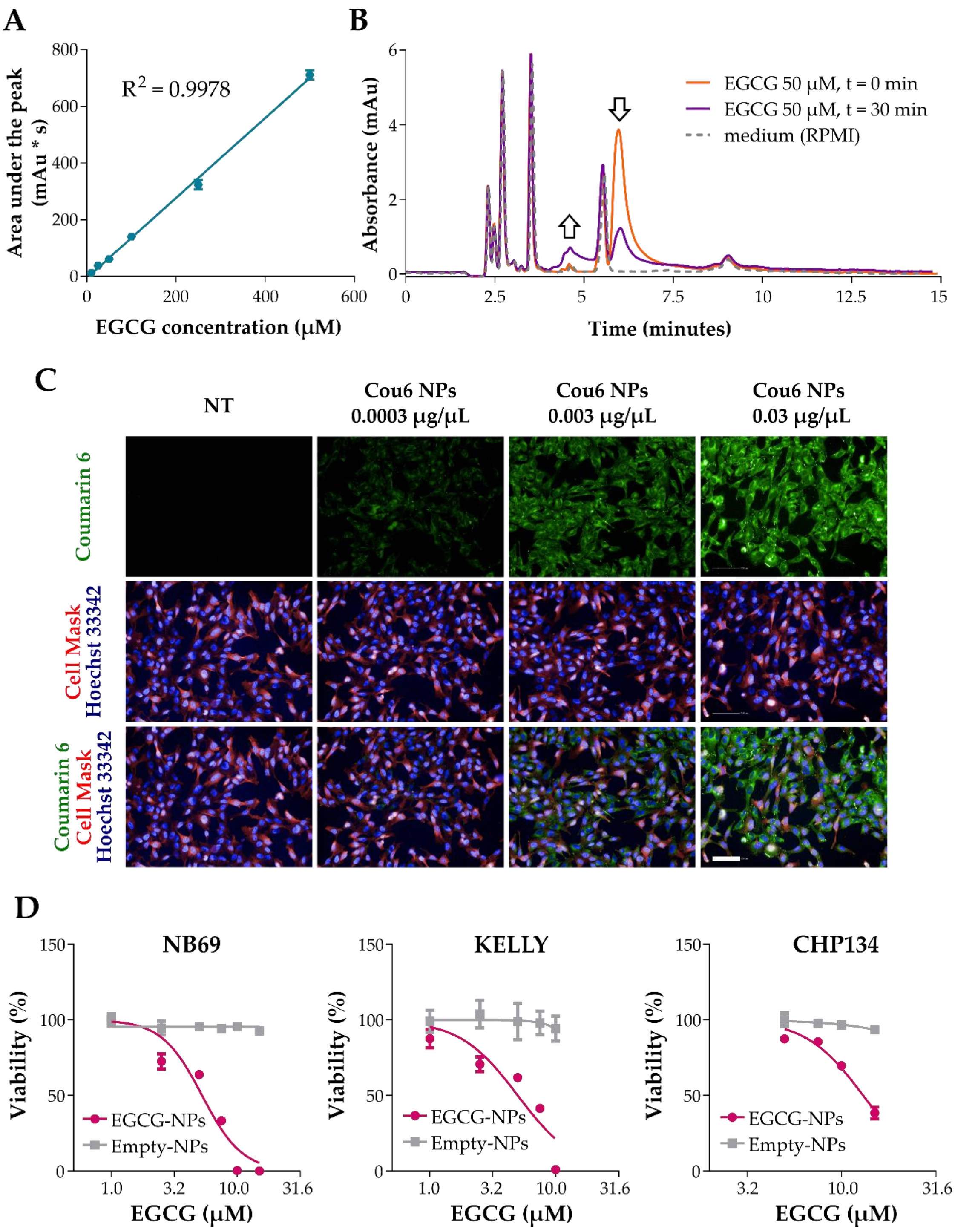

Figure S2. EGCG stability evaluation by HPLC and assessment of EGCG-NP penetration and effect in NB cells. (**A**) EGCG calibration curve. A series of EGCG solutions with different known concentrations (10 μM, 25 μM, 50 μM, 100 μM, 250 μM, 500 μM) was injected in an Agilent 1200 High HPLC system. A Phenomenex Gemini 5 μm C18 110 Å column was used in isocratic conditions with 80:20 water:acetonitrile with 0.01% TFA (pH 4-4.5). The flow rate and the detection were set at 1 mL·min^{-1}, and at 280 nm, respectively. n = 3 replicates, mean ± SD. R^2 = 0.9978. (**B**) Representative HPLC chromatogram of EGCG (50 μM) in RPMI-1640 at t = 0 and 30 minutes. EGCG retention time is 5.9-6.0 min. The chromatogram of the EGCG solution injected at t = 0 min is depicted in orange, whereas that of the EGCG solution at t = 30 min is depicted in purple. Cell culture medium (RPMI-1640) signals remain constant over time (grey). Arrows indicate EGCG instability in aqueous media

and the formation of degradation products. n = 3 replicates. (**C**) Representative immunofluorescence images of NB69 cells treated with different amounts of Cou6-NPs (green). Nuclei were stained with Hoechst 33342 (blue) and the cytoplasm with the CellMask™ Deep Red Stain (red). Images were acquired using the Operetta-High Content Imaging System. Scale bar = 100 µm. (**D**) Representative dose-response curves for EGCG-NPs and empty-NPs on three NB cell lines (NB69, KELLY, CHP134). After 48 h of treatment, cell viability was measured using the CellTiter-Glo® Luminescent Cell Viability Assay. Data are expressed as the percentage of viability normalized on non-treated cells. The graph shows one representative result of n = 3 biological replicates, n = 3 technical replicates each.

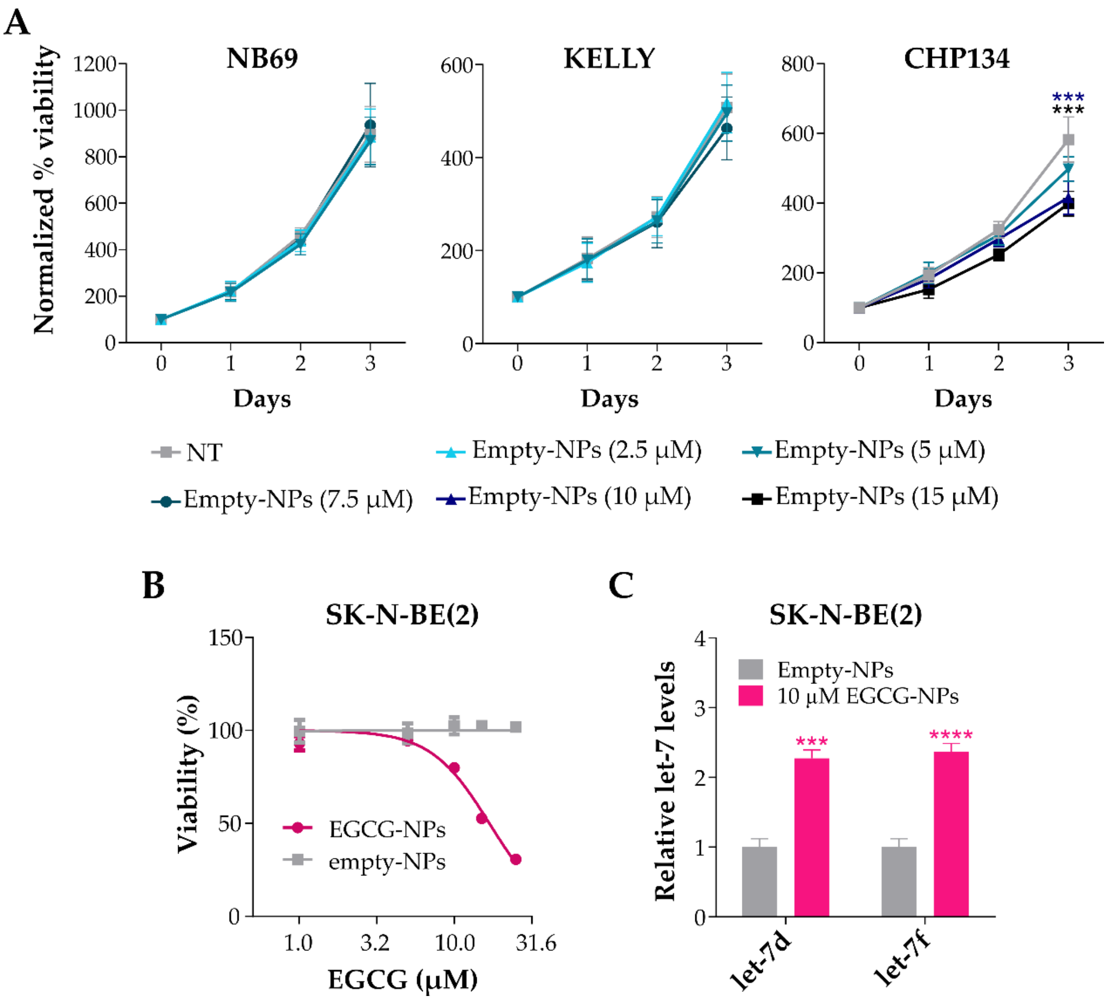

Figure S3. Empty-NP treatment only slightly affects NB cell proliferation, and EGCG-NP treatment increases let-7 miRNA levels in SK-N-BE(2) cells. (**A**) Proliferation curves of NB69, KELLY and CHP134 cells treated with several doses of empty-NPs corresponding to the doses of EGCG-NPs used in Figure 5B. Cell viability was measured using the CellTiter-Glo® Luminescent Cell Viability Assay and normalized on the day of the treatment (day 0). NT = non-treated cells. n = 3 biological replicates, n = 3 technical replicates each. Mean ± SEM. Two-way ANOVA followed by Fisher's LSD test (*** $p < 0.001$). (**B**) Representative dose-response curves for EGCG-NPs and empty-NPs in SK-N-BE(2) cell line. After 48 h of treatment, cell viability was measured using the CellTiter-Glo® Luminescent Cell Viability Assay. Data are expressed as the percentage of viability normalized on non-treated cells. A representative result is shown, n = 2 biological replicates, n = 3 technical replicates each. Mean ± SD. (**C**) qPCR analysis of let7-f and let7-d miRNAs levels in SK-N-BE(2) cells treated with empty-NPs or EGCG-NPs. Expression level is shown as fold change relative to empty-NPs, data were normalised on U6 internal reference gene. n = 3 technical replicates. Mean ± SD. Unpaired two-tailed t-test analysis (*** $p < 0.001$, **** $p < 0.0001$).

Table S2. Primer sequences

Gene	Forward	Reverse
**LIN28B**	5′ GAGTCAATACGGGTAACAGGAC 3′	5′CACCACAGTTGTAGCATCTATCT 3′
**SOX2**	5′ GTATCAGGAGTTGTCAAGGCAGAG 3′	5′ CTAGTCTTAAAGAGGCAGCAAAC 3′
**SOX9**	5′ GTACCCGCACTTGCACAAC 3′	5′ TCTCGCTCTCGTTCAGAAGTC 3′
**GAP43**	5′ GGCCGCAACCAAAATTCAGG 3′	5′CGGCAGTAGTGGTGCCTTC 3′
**TUBB3**	5′ TCAGCGTCTACTACAACGAGGC 3′	5′ GCCTGAAGAGATGTCCAAAGGC 3′
**NESTIN**	5′ CCTCAAGATGTCCCTCAGCC 3′	5′ TCCAGCTTGGGGTCCTGAAA 3′
**MYCN**	5′ CCGGGCATGATCTGCAA 3′	5′ CCGCCGAAGTAGAAGTCATCTT 3′
**TH**	5′ TCATCACCTGGTCACCAAGTT 3′	5′ GGTCGCCGTGCCTGTACT 3′
**HPRT1**	5′ TGACACTGGCAAAACAATGCA 3′	5′ GGTCCTTTTCACCAGCAAGCT 3′
**SDHA**	5′ TGGGAACAAGAGGGCATCTG 3′	5′ CCACCACTGCATCAAATTCATG 3′

Table S3. Protein purification: buffers composition.

Buffer Name	Composition
**Equilibration buffer (EQ)**	25 mM Tris-Cl pH 8; 100 mM NaCl; 0.05% NP-40; 1 mM DTT; 3 mM $MgCl_2$; 10 mM imidazole; Protease inhibitors (diluted 1:100)
**Wash1 (W1)**	25 mM Tris-Cl pH 8; 200 mM NaCl; 0.05% NP-40; 1 mM DTT; 3 mM $MgCl_2$; 50 mM imidazole.
**Wash2 (W2)**	25 mM Tris-Cl pH 8; 300 mM NaCl; 0.05% NP-40; 1 mM DTT; 3 mM $MgCl_2$; 50 mM imidazole
**Wash3 (W3)**	25 mM Tris-Cl pH 8; 300 mM NaCl; 0.05% NP-40; 1 mM DTT; 3 mM; $MgCl_2$; 70 mM imidazole
**Wash4 (W4)**	25 mM Tris-Cl pH 7; 500 mM NaCl; 0.05% NP-40; 1 mM DTT; 3 mM $MgCl_2$; 70 mM Imidazole
**Elution buffer (EL)**	25 mM Tris-Cl pH 8; 250 mM NaCl; 3 mM $MgCl_2$; 400 mM imidazole
**Dialysis buffer**	25 mM HEPES pH 7.4; 110 mM KCl; 10Mm NaCl; 1 mM $MgCl_2$; 15 µM $ZnCl_2$; 0.02% Tween; 0.1 % p/v ultrapure BSA
**Storage buffer (S)**	25 mM HEPES pH 7.4; 110 mM KCl; 10Mm NaCl; 1 mM $MgCl_2$; 15 µM $ZnCl_2$; 0.02% Tween; 0.1 % p/v ultrapure BSA, 15% glycerol, pH 7.5

The purification buffers were prepared in DEPC water.
